# Innovative Skin Product Emulsions with Enhanced Antioxidant, Antimicrobial and UV Protection Properties Containing Nanoparticles of Pure and Modified Chitosan with Encapsulated Fresh Pomegranate Juice

**DOI:** 10.3390/polym12071542

**Published:** 2020-07-12

**Authors:** Nikolaos D. Bikiaris, Georgia Michailidou, Maria Lazaridou, Evi Christodoulou, Eleni Gounari, Anna Ofrydopoulou, Dimitra A. Lambropoulou, Souzan Vergkizi-Nikolakaki, Smaro Lykidou, Nikolaos Nikolaidis

**Affiliations:** 1 Laboratory of Chemistry and Technology of Polymers and Dyes, Department of Chemistry, Aristotle University of Thessaloniki, 54124 Thessaloniki, Greece; mpikiaris@chem.auth.gr (N.D.B.); michailidougeorgia18@gmail.com (G.M.); marlazach@chem.auth.gr (M.L.); evicius@gmail.com (E.C.); egounari@biohellenika.gr (E.G); slykidou@chem.auth.gr (S.L.); 2 Laboratory of Environmental Pollution Control, Department of Chemistry, Aristotle University of Thessaloniki, 54124 Thessaloniki, Greece; anofr1991@gmail.com (A.O.); dlambro@chem.auth.gr (D.A.L.); 3 Department of Microbiology, School of Medicine, Faculty of Health Sciences, Aristotle University of Thessaloniki, 54124 Thessaloniki, Greece; sverg@auth.gr

**Keywords:** polysaccharide, chitosan derivatives, nanoparticles, pomegranate juice, cosmetic emulsions, antioxidant stability, antimicrobial properties, UV protection

## Abstract

In the present study, a chitosan (CS) derivative with the 2-(Methacryloyloxy)ethyl]dimethyl-(3-sulfopropyl)ammonium hydroxide (SDAEM) zwitterionic monomer was prepared through chemical modification. The successful synthesis of CS-SDAEM was confirmed by Fourier-transform Infrared (FTIR) and Nuclear Magnetic Resonance (^1^H-NMR) spectroscopies. Its crystallinity was studied by X-ray Diffraction (XRD), while in vitro cytotoxicity and cell viability assays established its biocompatibility. Filtered fresh pomegranate juice (PJ) was loaded in nanoparticles of neat CS and its derivative via ionic gelation method. Dynamic Light Scattering (DLS) revealed nanoparticles sizes varying between 426 nm and 4.5 μm, indicating a size-dependence on the polymer concentration used during encapsulation. High-performance liquid chromatography coupled with photodiode array and electrospray ionization mass spectrometry detection (LC-PDA-ESI/MS) revealed that PJ active compounds were successfully and in sufficient amounts encapsulated in the nanoparticles interior, whereas XRD indicated a crystalline structure alteration after nanoencapsulation. The resulted PJ-loaded nanoparticles were further utilized for the preparation of innovative O/W cosmetic emulsions. All produced emulsions exhibited good pH and viscosity stability for up to 90 days, while the sun protection factor (SPF) was enhanced due to the presence of the PJ. Enhanced antioxidant and antimicrobial properties due to the phenolic compounds of PJ were also observed.

## 1. Introduction

Skin is the largest organ of the human body and is continuously exposed to oxidative stress caused by the ultraviolet (UV) radiation or due to endogenous factors, namely enzymes [1]. Its proper hydration is of great importance for a non-flaky, healthy skin appearance [2]. Basic everyday skin care is mostly done through emulsions whose ultimate purpose is the increase of the water content located in the outermost layer of the epidermis, stratum corneum [3]. Emulsion products are used worldwide in the cosmetic and pharmaceutical industry for numerous applications besides skin moisturization, including cosmetic products and the dermal or transdermal drug delivery of various active pharmaceutical ingredients (APIs). Their chemistry has been therefore examined thoroughly.

An important factor for cosmetic emulsions is their antioxidant stability. It is of high significance since the intake of antioxidants could support the maintenance of good skin health and the prevention of oxidative stress. There has been an ongoing extensive research on artificial antioxidants which unfortunately demonstrated dose-dependent toxicological effects. Consequently, the search for new natural-derived antioxidants is crucial [4]. Various compounds have been applied as antioxidants for skin treatment products, mainly antioxidant drugs e.g., kojic acid [5], many vitamins i.e., Vitamin E (tocopherol) [6], Vitamin B3 [7], as well as several natural antioxidants namely lycopene which is a carotenoid [8], polyphenols derived from green tea [9], antioxidants extracted from the fruit of the coffee plant Coffea arabica [10] and proanthocyanidins extracted from Grape seed [11]. 

Pomegranate (*Punica granatum*), is an extremely interesting source of antioxidants; thus, it has been investigated thoroughly. It is a fruit derived from a shrub or a small tree (3–5 m height) which is well known since ancient times as Indian, Greek and Cuban civilizations used it to treat various diseases [12]. The edible part of the fruit is almost half of its total weight while the rest is peel [13]. The latter is composed of phenolic compounds, (flavonoids, hydrolysable tannins, organic and phenolic acids) [14] whereas, pomegranate juice (PJ) consists of 85% water and 10% sugars, anthocyanins, gallotannins, hydroxybenzoic acids, hydroxycinnamic acids, and dihydroflavonols [15]. The chemical composition of the fruit depends on a variety of factors namely the location of the cultivation, the climate or the degree of ripeness. The quality of the produced PJ is dependent on the production procedure as the ingredients of the final PJ are different when PJ is produced from the fleshy seed coat (sarcotestas) or from the whole fruit [13]. In general, phenolic compounds appear in a wide variety of structures ranging from simple aromatic compounds to complex polymeric chains [16]. Polyphenols, are the main responsible compounds for the high antioxidant activity of PJ [15], whereas, anthocyanins seem to have a photoprotective effect during photooxidative stress [17]. These compounds are capable of interfering with oxygen free radical generation and the reactions they subsequently trigger [5]. 

PJ has also high antibacterial and antimicrobial activities [18,19,20,21]. However, as already mentioned previously, it mainly contains water and thus high amounts of PJ should be added in cosmetic emulsions in order to ensure their activity. In a recent study, it was shown that fresh PJ at 20% had a minimum inhibitory concentration (MIC) equal to 100% (MIC_100%_) on 60 strains of *Staphylococcus epidermidis* that were tested. [22]. Therefore, a higher volume is necessary to be added in order to achieve substantial antimicrobial properties, which is not possible because of the large water content (80–90%) [23], unless juice concentrates or extracts are obtained. To avoid this, many groups have tried to encapsulate PJ and its active ingredients into polymeric matrices and use these materials to improve the antioxidant and antibacterial activities of the final products.

Arabestani et al. utilized pomegranate juice as an antioxidant source in protein-based films intended for active packaging [24] whereas, Yekdane et al. encapsulated pomegranate seed oil in Arabic and xanthan gum by spray drying using pomegranate juice as a wall material [25]. Also, Santiago et al. encapsulated PJ in various polymeric carriers (gum Arabic, modified starch, maltodextrin) by spray drying aiming to protect the anthocyanins of the PJ [26], while Robert et al., encapsulated PJ and its ethanolic extract in maltodextrin and soybean proteins microcapsules via spray drying and further added them into yogurt, revealing promising results [27].

In the present work, chitosan (CS) was chosen for nanoencapsulation of PJ in order to use these nanoparticles in cosmetic products. CS is a natural derived polymer with antimicrobial and antibacterial activity, low toxicity, biocompatibility and non-allergenicity [28]. It easily forms into nanoparticles through ionic gelation technique, a method for formulating nanoparticles through mild conditions. Nanoparticles are formed spontaneously through ionic interactions between the protonated amino groups of chitosan with the anionic groups of polyanions. Over the last years, nanoparticles have been utilized as potent carriers of various pharmaceutical drugs and cosmetic active ingredients [29]. More specifically, chitosan has been extensively studied for various applications namely drug nano-encapsulation for ocular release [30], COPD treatment [31], patches [32] and porous dressings [33] for sustained release, as well as encapsulation of annatto and saffron for the preparation of UV protective cosmetic emulsions [34].

**Figure 1:**
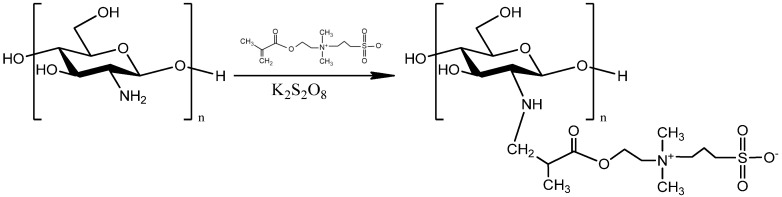
Chemical modification of chitosan with 2-(methacryloyloxy)ethyl]dimethyl-(3-sulfopropyl)ammonium (SDAEM).

Even though chitosan demonstrates numerous advantages as a polymer, the chemical modification of its structure is an effective way to introduce new functional groups in its macromolecular chains. It is a useful method to ameliorate the chemical properties and to prepare new materials with potential uses in different applications. Zwitterions are compounds which contain positively and negatively charged groups resulting in an overall neutral charge [35]. As materials, they are able to reduce non-specific protein adsorption as well as bacterial adhesion, and provide an increased bioavailability [36]. Furthermore, zwitterionic homopolymers are capable of delaying or even preventing microbial attachment to surfaces [37]. 2-(Methacryloyloxy)ethyl]dimethyl-(3-sulfopropyl)ammonium hydroxide (SDAEM) (Figure 1) is a hydroscopic zwitterionic monomer, which was utilized in the present study in order to further increase the antibacterial and antimicrobial properties of the polysaccharide. 

According to all that described above, the aim of the present study was the modification of CS with the zwitterionic monomer SDAEM and the preparation of CS and CS-SDAEM nanoparticles with PJ encapsulated in their interior. Furthermore, the eventual intention is the assessment of the applicability of these nanoparticles in innovative O/W cosmetic emulsions, and the estimation of their contribution to the antioxidant, sun protective and antimicrobial properties of the emulsions. Such a study is reported in literature for the first time. 

## 2. Materials and Methods 

### 2.1. Materials

Chitosan with molecular weight 18000 g/mol, and a degree of deacetylation > 94% as described in a previous paper of our group [31], was supplied by Kraeber &Co GmbH (Ellerbek, Germany). Sodium tripolyphosphate (TPP) used as the ionic cross-linker for the preparation of the nanoparticles was supplied from Aldrich chemicals (St. Louis, Missouri, United States) and was of 85% *w*/*w* purity. The monomer 2-(methacryloyloxy)ethyl]dimethyl-(3-sulfopropyl)ammonium (SDAEM) was of 95% purity and was supplied from Fluorochem Ltd. (Hadfield, Glossop, UK). Fresh, biologically produced and mechanically squeezed under cold conditions, pomegranate juice, was kindly donated from Vioagros SA (Thessaloniki, Greece). Olive oil, ethylhexylglycerin, shea butter, glycerin, cetostearyl alcohol, cetyl alcohol, sodium citrate, beeswax, xanthan gum, polysorbate 60, stearic acid, and phenoxyethanol with appropriate purity for cosmetic emulsions, were kindly donated from Novita Group (Thessaloniki, Greece). All other materials used in the present study were of analytical grade.

### 2.2. Chemical Modification of CS

CS structure was modified with 2-(methacryloyloxy)ethyl]dimethyl-(3-sulfopropyl) ammonium (SDAEM) (Figure 1). More specifically, 10 g of CS powder were dissolved in 500 mL acetic acid solution 2% *v*/*v* under stirring and 1.5 g of the monomer SDAEM was added. Potassium persulfate was utilized as the initiator of the reaction and 4.8 mg of this were added to the above solution and mechanical stirring was conducted for 12 h. Following this, 180 mL of NaOH 4 N were added to the solution so as to precipitate the polymer. The resulted modified polymer was freeze-dried and consequently treated with Soxhlet extraction using methanol as solvent in order to remove any unreacted SDAEM monomer. 

### 2.3. Preparation of the PJ-Encapsulated Nanoparticles

The nanoparticles were prepared according to the long-established ionic gelation technique using sodium tripolyphosphate (TPP) as the polyanion [38]. According to Koukaras et al., the optimum weight ratio between CS and TPP was 4/1 [38]. The innovation of the present study was the alternation of the concentration of the polymeric solutions. More specifically, proper amount of CS was dissolved in aqueous acetic acid solution (pH about 4.5) in order to provide final polymeric solutions with CS concentrations of 2.5% *w*/*v*, 5% *w*/*v*, 7.5% *w*/*v* and 10% *w*/*v*. Pomegranate juice was filtered under vacuum with a series of Whatman filters ranging from No 1 to No 5 that differed in the degree of particle size retention from coarse particles of 25 μm to very small particles of 2.5 μm, in order to remove all the non-edible parts of white pith and the seeds. The filtration listed above laboriously produced pomegranate juice of strongly dark red clear solution free of any turbidity or sediments. For PJ encapsulation the filtered liquid was added to the different CS concentrations in a constant volume ratio 1:1 (10 mL PJ volume to 10 mL volume of the CS solution. TPP aqueous solutions of different TPP concentrations (6.25 mg/mL, 12.50 mg/mL, 18.75 mg/mL and 25 mg/mL) were also prepared in order to maintain the final weight ratio among CS and TPP constant and equal to 4/1. These TPP solutions were added dropwise to the homogenous solutions of CS and pomegranate juice under magnetic stirring. The encapsulation of the juice occurred spontaneously when the TPP solution was added, which was also visually proved since the formed nanoparticles were colored red as PJ and these mixtures have been named as CS-TPP-PJ. Exactly the same procedure was followed for the encapsulation of PJ in CS-SDAEM nanoparticles using polymeric solutions 2.5% *w*/*v*, 5% *w*/*v*. 7.5% *w*/*v* and 10% *w*/*v* and these materials have been named as CS-SDAEM-TPP-PJ. 

### 2.4. Characterization of the Polymer and the Nanoparticles

#### 2.4.1. Fourier-Transform Infrared Spectroscopy (FTIR)

The FTIR spectra of the samples were obtained by FTIR spectrometer (model FTIR-2000, Perkin Elmer, Waltham, MA, USA). Briefly, a small amount of each sample was triturated with a proper amount of potassium bromide (KBr) and the disks were formed under pressure. The spectra were collected in the range from 400 to 4000 cm^−1^ at a resolution of 4 cm^−1^ using 16 co-added scans and the baseline was corrected and converted into absorbance mode.

#### 2.4.2. ^1^HNMR

^1^H NMR spectra of pure CS and its CS-SDAEM derivatives were recorded on an Agilent 500 spectrometer (Agilent, Santa Clara, CA, USA), at a frequency of 500 MHz. The polymer was dissolved in deuterated acetic acid solution (CD3COOD 2% *v*/*v*). The measurement was conducted at room temperature, relaxation delay was 1 s and the number of scans was 16. Spectra were internally referenced with tetramethylsilane (TMS) and calibrated using the residual solvent peaks. 

#### 2.4.3. Wide angle X-Ray Scattering (XRD)

X-ray powder diffraction (XRD) patterns were recorded using an XRD-diffractometer (Rigaku-Miniflex II, (Rigaku, Austin, TX, USA) with a CuKα radiation for crystalline phase identification (λ = 0.15405 nm). The sample was scanned at the range of 5 to 50° with a scan speed 1°/min.

#### 2.4.4. In Vitro Cytotoxicity Studies

The cytotoxicity of CS, SDAEM and CS-SDAEM was evaluated by measuring the viability of Adipose tissue-derived mesenchymal stem cells (ASC) in the presence of the studied materials. ASCs were used due to their immediate attachment on different materials surfaces and also their high proliferation capacity, aiming the reliable determination of cytotoxicity. The viability of the ASC cells was tested through [3-(4,5-dimethylthiazol-2-yl)-2,5-diphenyl tetrazolium bromide] (MTT) assay. The tested materials were cut in squares with dimensions 9 × 9 mm and were sterilized under decreasing concentrations of ethanol (100%-70%-50%), washed with distilled water and dried for 4 h under sterile conditions. Then, they were placed in the bottom of the 24-well plate with fibrin glue. The cells were seeded in these 24-well plates at a density of 300.000 cells per well, in 1 mL cell culture medium, whereas the control sample was constituted from cells plated directly on the plate. After 24 h of cell’s incubation in the culture medium, the medium was removed and fresh culture medium solution containing MTT was added. After 4 h cultured in 37 °C at 5% CO_2_ atmosphere, the MTT solution was removed, 1 mL/well DMSO was added and the incubation in the same conditions carried out for 1 h. The reduction of MTT was estimated with a UV spectrometer (Perkin Elmer, Waltham, MA, USA) in 570 nm and 630 nm. 

#### 2.4.5. Dynamic Light Scattering (DLS)

The size of the nanoparticles (100 μL of the suspended nanoparticles were dispersed in 900 μL of double distilled water) as well as their zeta potential were determined by dynamic light scattering (Zetasizer 5000, Malvern Instruments, Worcestershire, United Kingdom). All measurements were performed in triplicate.

#### 2.4.6. Liquid Chromatography-Mass Spectroscopy (LC-ESI-MS)

The identification of the organic compounds of the PJ encapsulated in CS and its CS-SDAEM derivative as well as the determination of the encapsulation efficacy, was conducted with a liquid chromatograph with a photo diode array (PDA) detector coupled in series with a mass spectrometry (MS) detector (LCMS 2010EV, Shimadzu, Tokyo, Japan) equipped with an atmospheric pressure electrospray ionization source (ESI). For the analysis, the methods of Sentandreu et al. [39] and Mena et al. [40] were utilized. The HPLC system was equipped with a SIL20A autosampler and an LC-20AB pump both from Shimadzu (Kyoto, Japan). The utilized column was a CNW HPLC Athena C18 column, 4.6 mm × 250 mm and 5 μm pore size, while the mobile phase was MeOH (A) and H_2_O-HCOOH 0.1% (B) and the flow rate was set to 0.2 mL/min while the temperature was 40 °C. The injection volume was 20 μL while the gradient elution was 1 min 90% B, 5 min 80% B, 10 min 60% B, 20 min 40% B, 25 min 0% B and 30 min 0% B. MS measurements were carried out in positive and negative ionization mode. The drying gas was nitrogen operated at 10 L/min, 200 °C and the nebulizing pressure was 100 psi. The capillary voltage was set to 4500 V for the positive ionization and to −3500 V for the negative ionization. For the determination and quantification of the compounds, the protonated ion [M+H] and the deprotonated ion [M-H] were chosen as main molecular ions for the positive and negative determination respectively. 

### 2.5. Preparation of the Emulsions

The preparation of the emulsions was conducted according to O/W (oil in water) technique [34]. O/W emulsions were prepared containing nanoparticles prepared with CS-TPP-PJ 2.5% *w*/*v* and CS-SDAEM-TPP-PJ 2.5% *w*/*v* concentrations. Table 1 presents the utilized ingredients for the emulsions’ preparation. The water phase consisted of water (140 g, 70% *w*/*v*), glycerin (7 g, 3.5% *w*/*v*), xanthan gum (2 g, 1% *w*/*v*) and citric acid (1 g, 0.5% *w*/*v*). The water phase was heated in a water bath at 80 °C, under mechanical stirring until its homogenization and constituted 75% of the final emulsion. The oil phase consisted of olive oil (26 g, 13% *w*/*v*), cetyl alcohol (4 g, 2% *w*/*v*), cetostearyl alcohol (4 g, 2% *w*/*v*), polysorbate 60 (4 g, 2% *w*/*v*), shea butter (4 g, 2% *w*/*v*), steatic acid (4 g, 2% *w*/*v*), and beeswax (4 g, 2% *w*/*v*). The oil phase was heated in a water bath until the solution was completely dissolved and homogenous and constituted 25% of the final emulsion. Following this, 2.5 g of CS or CS-SDAEM were dissolved in 125 mL aqueous CH_3_COOH 2% *v*/*v* and 125 mL PJ, providing a final polymeric solution of 1% *w*/*v*. Nanoparticles were obtained spontaneously by adding 20 mL of TPP solution (6.25 mg/mL). Proper volume of the formulated CS-TPP-PJ 2.5% *w*/*v* and CS-SDAEM-TPP-PJ 2.5% *w*/*v* nanoparticles dispersions were homogenized in the water phase using probe sonication (100 W, 30 kHz, Hielscher Ultrasonics) for 2 min, in proper ratios with the intention of forming various nanoparticles concentrations (0.7, 1.4, 2.1, 2.9% *w*/*v*). The volume of the added nanoparticles was abstracted from the total water volume. Finally, for the preparation of the final emulsions, the oil phase was added to the water phase in O/W ratio 25/75, under continuous magnetic stirring and the O/W emulsions occurred spontaneously. The resultant O/W emulsions were left under magnetic stirring for 2 h and after which phenoxyethanol and ethylhexylglycerin were added. Finally, a control emulsion without the presence of PJ nanoparticles was also prepared according to the procedure mentioned above.

### 2.6. Characterization of the Emulsions

#### 2.6.1. Emulsion Stability

The stability of the produced emulsions was determined via pH and viscosity measurements after 1, 7, 14, 30, 60 and 90 days since the preparation of the emulsions. The pH value was assessed by dipping the pH sensor (Microprocessor, WTW, pH 535, Gemini BV, Apeldoorn, The Netherlands) in to the emulsions. Viscosity measurements were performed under 50 and 100 rpm using the R3 spindle of a Visco Star Plus viscometer.

#### 2.6.2. Centrifugation Tests

Centrifugal tests of the O/W emulsions were performed at 5000 rpm and 25 °C for 10 min, by placing 10 g of each sample in centrifugal tubes. The tests were performed directly after preparation as well as after 1, 3 and 7 days of storage.

#### 2.6.3. In Vitro Antibacterial Activity Testing 

The antibacterial activity of the synthesized derivatives (1.75 wt% in acetic acid solution) and emulsions was evaluated using agar well diffusion method. In brief, the inhibition zones were measured on Mueller Hinton agar plates inoculated with *Staphylococcus aureus* (*S. aureus*) and *Escherichia coli* (*E. coli*). For each of these bacteria, nutrient broth was first inoculated with isolated colonies of either bacteria and cultured at 37 °C for 18–24 h. 100 μL of this culture was further inoculated into Mueller Hinton Broth (Steinheim, Germany) and incubated for 2 h and the turbidity adjusted to 0.5 McFarland. 100 μL of this culture was then spread on Mueller Hinton agar plates, wells (4.6 mm diameter) were cut in to the agar with a sterile borer and 100 mg of each preparation was placed into each well. The plates were incubated at 37 °C for 24 h and diameters of the inhibition zone around each well were measured. A blank sample containing only nutrient broth and a positive sample containing Neomycin disc were also tested. Each test was repeated three times and it was noted that the differences were negligible.

#### 2.6.4. Antioxidant Activity

The antioxidant activity of the samples was determined with the 2,2-Diphenyil-1-picrylhydrazyl (DPPH) method, which was developed according to Blois in 1958 [41]. 1 mL of each emulsion dispersed in EtOH (1% *v*/*v*) was added to 3 mL of a 5 × 10^−3^ mg/mL ethanol DPPH solution. The reference sample composed of 1 mL EtOH and 3 mL of the DPPH/EtOH solution. The samples were sonicated and their absorbance was recorded 30 min later with the aid of a UV-Vis spectrometer (UV Probe 1650, Shimadzu, Tokyo, Japan) at 517 nm. The free radical scavenging activity was described as reported by Brand et al. according to the following equation [42].

(1) $$\mathrm{Free}\;\mathrm{radical}\;\mathrm{scavenging}\;\mathrm{activity}\;(\%)=\frac{Absorbance\;of\;control-Absorbance\;of\;extracts}{Absorbance\;of\;control}\;\times \;100$$

#### 2.6.5. SPF Determination

For the SPF determination, diluted solution transmittance method was utilized [43]. 1 g of each sample was weighted, transferred to a 100 mL volumetric flask, sonicated until complete homogenization, diluted to volume with ethanol, mixed for 5 min and filtered through Whatman filters. 2 mL of each sample were transferred to a 10 mL volumetric flask and diluted with ethanol. The absorption values of the samples were obtained in the range of 290–320 nm (every 5 nm) using a UV-Vis spectrophotometer (Shimadzu, Tokyo, Japan) while experiments for each sample were performed in triplicate. Mansur equation was used to determine the SPF values of the formulations.

(2) $$SPFin\;vitro=CF\times \overset{320}{\underset{290}{\sum }}EE\left(\lambda \right)\times I\left(\lambda \right)\times abs\;\left(\lambda \right)$$

where CF = 10 (correction factor), EE (λ) = erythemogenic effect of radiation at wavelength λ, I(λ) = intensity of solar light at wavelength λ, and abs(λ) = absorbance of sample at wavelength λ. The values for the term “EE × I” are constants, which were determined by Sayre et al. [44].

## 3. Results & Discussion

### 3.1. Characterization of Modified CS 

**Figure 2:**
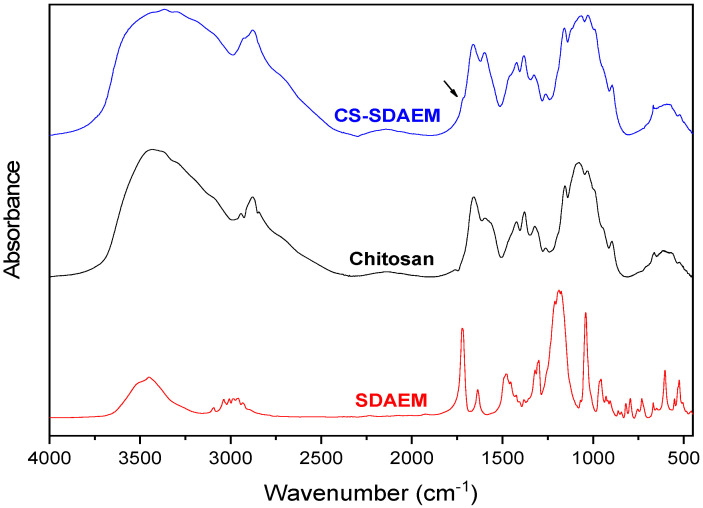
FTIR spectra of CS, SDAEM and CS-SDAEM derivative.

One of the primary purposes of the present study was the modification of the macromolecular chains of chitosan with a zwitterionic monomer in order to increase the amphiphilic character of the polysaccharide and thus its antimicrobial properties. This is a critical step in order to prepare cosmetic products with enhanced antimicrobial activity, along with antioxidant and UV protective properties, an objective that is being approached here. It is well known that CS has some antimicrobial activity [45,46] and that its quaternate derivatives exhibit much better antibacterial properties against several negative and gram-positive bacteria [47,48,49]. For this reason, SDAEM, containing a quaternate amino group, was utilized for the functionalization of CS, in a 6:1 molar ratio between CS and SDAEM. Figure 2 shows the FTIR spectra of pure CS, SDAEM and that of the modified derivative CS-SDAEM. The characteristic bands of CS spectra are present at 3400 cm^−1^ for O-H stretching, at 3280 cm^−1^ attributed to the -NH_2_ stretching and at 1656 cm^−1^ and 1584 cm^−1^ owing to the >C-O stretching (Amide I and II respectively) [50,51]. The characteristic bands of SDAEM are located at 3093 cm^−1^, 3038 cm^−1^ and 2988 cm^−1^ (C-H stretch), at 1715 cm^−1^ (C=O stretch), at 1644 cm^−1^ (C-N stretch vibration), at 1186 cm^−1^ and 1173 cm^−1^ (symmetrical and asymmetrical stretch of S=O bond) [52]. In the FTIR spectra of CS-SDAEM derivative, the successful modification of CS is indicated through the appearance of a new peak recorded as a shoulder at 1718 cm^−1^, which is attributed to the ester group of the monomer. Furthermore, the peak corresponded to the S=O group was also recorded in CS-SDAEM spectrum. However, this could not satisfactorily serve as an evidence since a small peak in the spectrum of neat CS is also recorded at the same area. For that reason, in order to further boost our initial assumptions and confirm the derivative synthesis, nuclear magnetic resonance spectroscopy (^1^H-NMR) was also applied.

**Figure 3:**
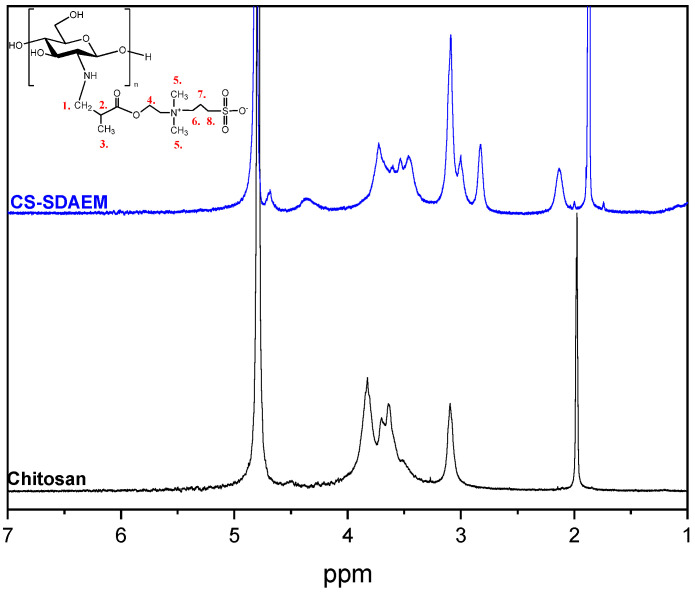
^1^H-NMR spectra of CS and CS-SDAEM derivative.

As can be seen in Figure 3**,** neat CS has several characteristic peaks. These peaks are also evident in the spectrum of CS-SDAEM derivative, where however some additional peaks were recorded as well. The peak at 1.97 ppm of neat CS corresponds to the methyl protons of the N-acetyl group of the polysaccharide [53]. This peak is shifted to 1.86 ppm in the CS-SDAEM spectrum due to the insertion of the SDAEM monomer onto the CS backbone. Furthermore, in the CS derivative’s proton NMR spectrum, there is an additional peak at 2.12 ppm, which matches the protons of the methylene group between the sulfonate and the quaternary amine groups. Also, the additional peak at 2.81 ppm corresponds to the methylene protons connected to the sulfonate group, whereas the rest of the methylene groups of SDAEM are present in the area between 3.5–4 ppm, but because of the occurring overlap of the peaks, it is difficult to distinguish them. Furthermore, the peak at 3.10 ppm corresponds to the six protons of the methyl groups, which belong to the quaternary amine of the monomer, while the new recorded peak at 4.65 ppm corresponds to the methyl group which is located next to the oxygen of the ester group of the monomer [52]. An interesting observation here is the absence of the peaks at 5.8 and 6.18 ppm which are witnessed in the spectrum of SDAEM. This absence of the double bond of the monomer is an indication that the reaction took place between the amino groups of CS and the double bond of the monomer. It is also a proof that there is no remained unreacted monomer and that the purification procedure using Soxhlet extraction was successful. 

**Figure 4:**
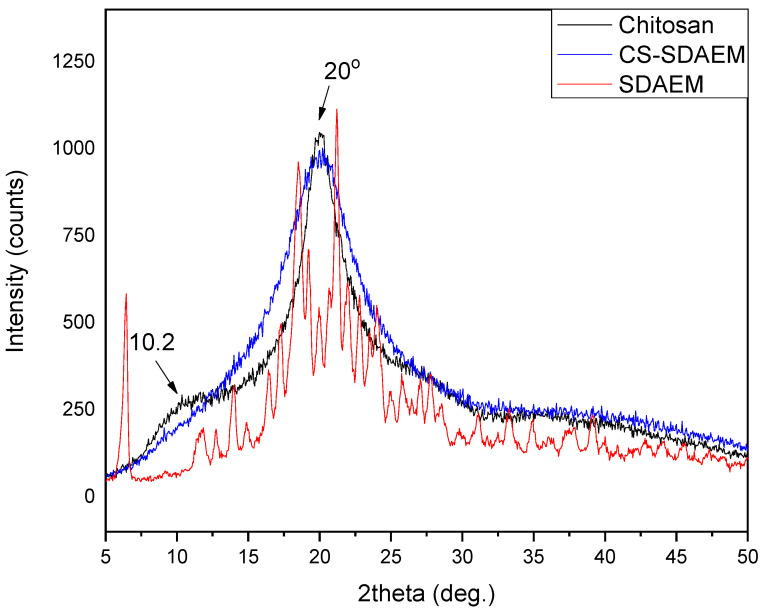
XRD patterns of neat CS and CS-SDAEM derivative.

In a subsequent stage, the crystal structure of the newly formed material was examined via XRD. CS is a semi-crystalline polymer which reveals two peaks at 10.2° and 20°. The results of the diffraction pattern of Figure 4 clearly indicate an alternation in the crystallinity of the CS-derivative compared to that of CS. More specifically, the peak at 10.2° disappeared completely, while the one situated at 20° became wider. This alternation is probably attributed to the introduction of the SDAEM monomer in the polymeric chains of CS. The presence of the monomer interrupts the formation of folds of the macromolecular chains and the following crystallites creation, which consequently reduces the degree of crystallinity of the CS-SDAEM derivative in comparison to neat CS. This reduction is in well agreement with previous reported findings in literature, concerning the synthesis of other CS derivatives and was attributed to the addition of bulkier side groups in CS macromolecule [54,55,56,57]. 

**Figure 5:**
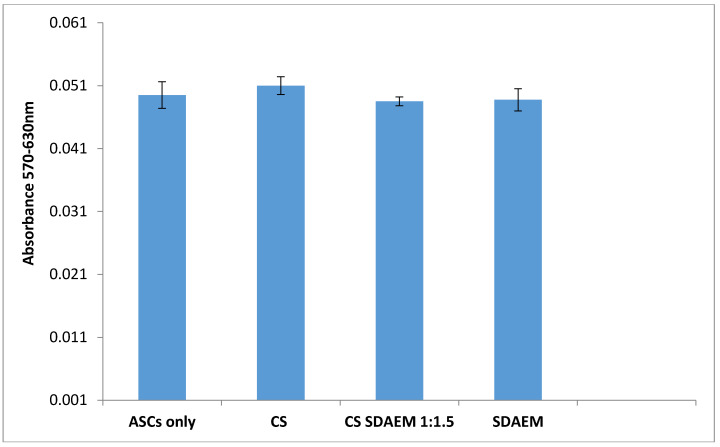
Cytotoxicity of CS, SDAEM and CS-SDAEM through MTT assay on adipose tissue-derived mesenchymal stem cells (ASC), UV absorbance of the samples.

Moving on, another important parameter for a material designed to be in contact to human skin, is its cytotoxicity. Concerning CS cytotoxicity, various studies have shown that it is an inert, non-toxic material while according to Huang et al., the cell uptake is dependent mainly on the degree of deacetylation of the polysaccharide [58]. The SDAEM monomer has been utilized extensively in the modification of various polymers namely poly(vinyl alcohol) [59] or modified beta-cyclodextrin [60] resulting in non-toxic materials, rendering it as a promising monomer concerning biocompatible applications. In the present study, the cytotoxicity of CS-SDAEM was evaluated via MTT assay which is based on the mitochondrial activity of the cells. The living cells are able to reduce MTT to water-insoluble formazan crystals which have purple color and can easily be evaluated via UV spectroscopy. Figure 5 presents the cytotoxicity results of CS, SDAEM monomer and CS-SDAEM derivative on adipose tissue-derived mesenchymal stem cells (ASC). The absorbance of the CS-SDAEM sample is similar to the monomer and neat CS. Consequently, the viability of the cells is not affected by the modification and thus, the CS-SDAEM derivative is a non-toxic material suitable for being in contact to the human skin. 

### 3.2. Characterization of Prepared PJ-Encapsulated Nanoparticles 

CS and CS-SDAEM nanoparticles with fresh pomegranate juice (PJ) encapsulated in their interior were formed via ionic gelation technique. It is a very simple technique capable of spontaneously forming nanoparticles through mild conditions. This method exploits the ability of CS, which is a polycationic polymer, to automatically form nanoparticles when interacting with sodium tripolyphosphate (TPP) which is utilized as a polyanion. Table 2 summarizes the results derived from DLS measurements concerning the size of prepared nanoparticles by using different CS and CS-SDAEM concentrations (2.5, 5, 7.5 and 10 wt%). This study has been carried out in order to estimate the optimum polymer concentration to produce nanoparticles of a certain size, since size was reported as a crucial factor concerning the chemical behavior of the formulated nanoparticles and the stability of the final product [61]. Moreover, because of the novelty of the formulation, there were no available data linking the polymer solution concentration and the amount of encapsulated PJ with the particle size, and thus DLS measurements would provide a significant insight. 

From Table 2 it is concluded that by increasing the polymer concentration, a larger final size of nanoparticles is obtained. More specifically, concerning the CS nanoparticles, CS-TPP-PJ 2.5 wt% sample’s size was measured at 647 nm. Interestingly, all higher polymer concentrations, resulted in particles in micro-scale, revealing a dramatic and relatively proportional increase of their size. This is in well agreement with Jonassen et al., who have found that by increasing CS concentration, nanoparticles of larger size have been formed, while the same effect is being observed by the increase of the ratio between the polymer and the polyanion [62]. On the contrary, concerning the CS-SDAEM material, all formed nanoparticles are of smaller size, while their diameter again rises by increasing the polymer concentration. As already described above, the SDAEM monomer contains both positive and negative charges and therefore, the emerged ionic interactions between the CS-SDAEM chains lead to nanoparticles of smaller size. These data are in well agreement to the results from a previous study from our group [30]. In that case, the modification of CS with succinic anhydride and 2-carboxybenzaldehyde results in smaller size particles, compared to CS nanoparticles [30]. This is an additional advantage of CS-SDAEM derivative compared to neat CS. Nevertheless, the optimum concentration, where the minimum sized nanoparticles are produced, are the samples CS-TPP-PJ 2.5 wt% and CS-SDAEM-TPP-PJ 2.5 wt%. 

Furthermore, the samples were evaluated concerning their zeta potential. Zeta potential describes the surface charge of the nanoparticle. According to Clogston and Patri, nanoparticles with zeta potential values greater than +30 mV or less than −30 mV are considered as strongly cationic and anionic respectively, and consequently capable of forming steady emulsions [63]. The same observation was done by Abouelhag et al., but this group defined as steady suspensions those with zeta potential values greater than +25 mV or less than −25 mV respectively [64]. In literature, CS nanoparticles derived via ionic gelation technique naturally reveal positive zeta potential values [65]. In the present study, the resulted nanoparticles are all positively charged. Interesting is the fact that while raising the concentration of the polymer (CS or CS-SDAEM), an increase of the zeta potential value of the formulated nanoparticles is observed. This observation is in agreement with literature, as the larger amount of polymer results in an increase of the free amino groups, which contribute to the enhanced positive charge [66].

**Figure 6:**
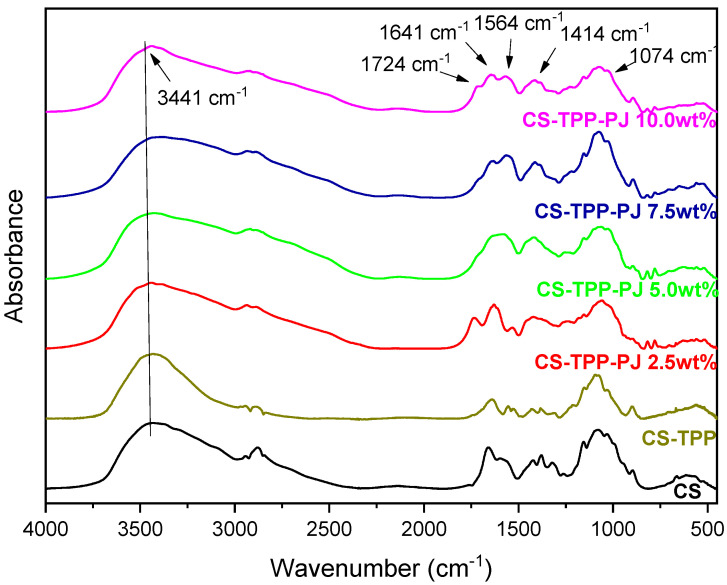
FTIR spectra of CS nanoparticles 2.5, 5.0, 7.5 and 10.0 wt% containing PJ.

**Figure 7:**
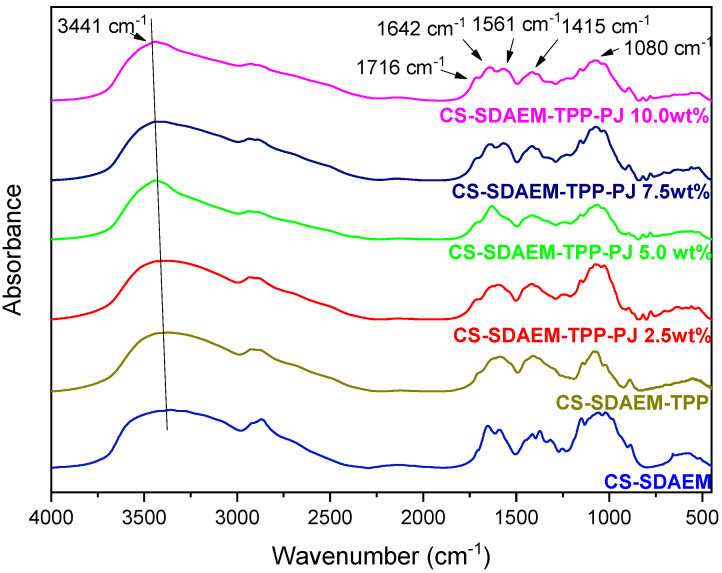
FTIR spectra of CS-SDAEM nanoparticles 2.5, 5.0, 7.5 and 10.0 wt% containing PJ.

FTIR analysis was conducted with the aim to evaluate the successful encapsulation of PJ into the nanoparticles, as well as to estimate any possible interactions between CS or CS-SDAEM with the active compound [67]. Figure 6 and Figure 7 depict the FTIR spectra of pure CS and CS-SDEM along with their formulated PJ-encapsulated nanoparticles. Concerning the CS nanoparticles, the presence of a new peak at 1724 cm^−1^, is attributed solely to the presence of the PJ, since neat CS has no absorbance peaks in that area, indicating a successful insertion of the PJ. The majority of the compounds which are present in the PJ, include carboxyl and hydroxyl groups in their molecular structure. Consequently, the absorbance of the hydroxyl groups in the nanoparticles’ spectra is shifted from 3436 cm^−1^ to 3441 cm^−1^ due to the PJ compounds. Additionally, the detected characteristic peaks between 1250–1500 cm^−1^ and 1042–1178 cm^−1^ are also ascribed to the presence of the PJ. The interactions between the polymeric matrix and the PJ are verified with the shift of the already existing absorbance peaks or the appearance of new ones. Moreover, the peak present at 1657 cm^−1^ corresponding to amide I of CS, is shifted to 1645 cm^−1^, attributed to interactions between CS amino groups and the phenolic groups of anthocyanins present at the PJ. This result is in agreement to the results of Tan et al. [68] where the encapsulation of anthocyanins in CS and chondroitin sulfate polyelectrolyte complexes occured. In the CS-SDAEM nanoparticles a shift of the carbonyl group of the modified material from 1721 cm^−1^ to 1716 cm^−1^ is observed, suggesting the presence of hydrogen bonds between the PJ compounds and the carbonyl groups of the modified CS. All in all, it is clear that PJ was successfully encapsulated into the prepared nanoparticles, a fact that was also evident with naked eye since all nanoparticles got the colour of PJ. Also, it is evident that some interactions are taking place between the reactive groups of PJ (-OH and -COOH) and the functional groups of CS and its derivative. These interactions are of great importance as was accentuated by Patel et al. [69], where they examined the interactions after the encapsulation of anthocyanins into soy protein and jackfruit seed starch, two natural polymeric materials, indicating serious variations in the antioxidant activity of the samples.

**Figure 8:**
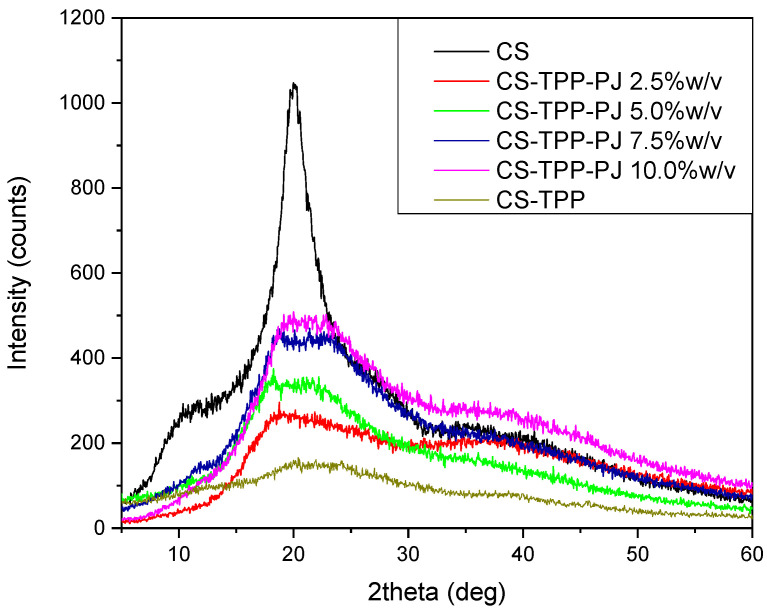
XRD patterns of CS, CS empty nanoparticles and CS nanoparticles 2.5, 5.0, 7.5 and 10.0 wt% containing PJ.

**Figure 9:**
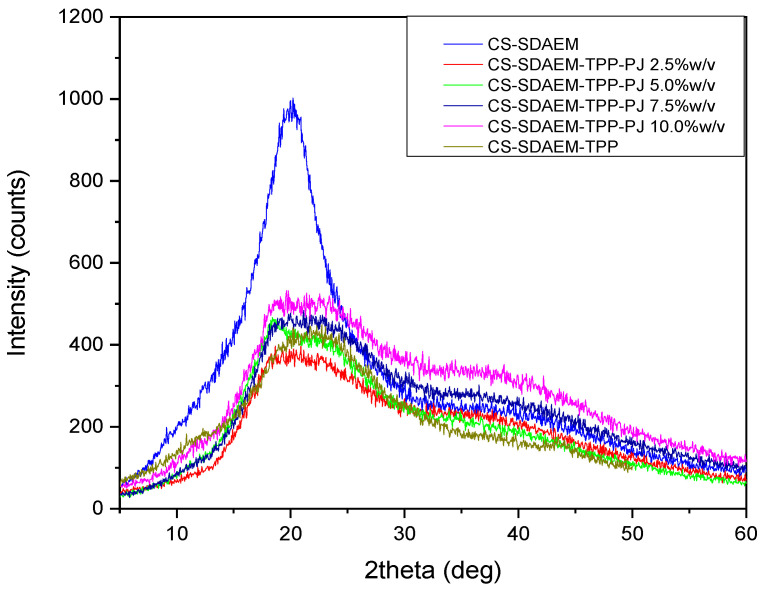
XRD patterns of CS-SDAEM, CS-SDAEM empty nanoparticles and CS-SDAEM nanoparticles 2.5, 5.0, 7.5 and 10.0 wt% containing PJ.

X-ray diffraction analysis was conducted with the intention to assess the physical state of PJ-encapsulated nanoparticles. As mentioned above, CS is a semi-crystalline polymer with two characteristic crystalline peaks at 10° and 20° [70], whereas the CS-SDAEM derivative exhibits only the broader peak at 20°. Figure 8 and Figure 9 present the XRD spectra of CS-TPP-PJ and CS-SDAEM-TPP-PJ nanoparticles. As evidenced, the formulation of nanoparticles through the ionic gelation method and the subsequent encapsulation of PJ results in an alternation of the crystalline structure of the prepared nanoparticles. It is obvious that the intensity of the recorded patterns and thus the crystallinity of the prepared nanoparticles is reduced, compared to the initial polymeric materials. Furthermore, it is clear that the intensity is directly depended on the used polymer concentration. This reduction is attributed to the different arrangement of the polymeric chains after the interaction with the polyanion during the formation of the nanoparticles. Moreover, the intensity of the peak at 10° is decreased while the peak at 20° is divided in two different peaks. This alternation is attributed to the different crystallization of CS or CS-SDAEM due to the formation of the nanoparticles or due to the presence of the PJ. Similar results have been reported in our previous study by encapsulating a drug in CS derivatives, indicating that nanoparticles with lower crystallinity have been prepared [30]. 

In a further step, LC-ESI-MS with positive (ESI[+]) and negative (ESI[–]) ionization conditions, was utilized with the intention to identify the chemical compounds which are present in the PJ as well as to determine the encapsulation percentage of these compounds in the prepared nanoparticles. It was applied in samples CS-TPP-PJ 2.5 wt%, CS-TPP-PJ 5.0 wt%, CS-SDAEM-TPP-PJ 2.5 wt% and CS-SDAEM-TPP-PJ 5.0 wt%, since they scored the finest results concerning the nanoparticle size measurements. The encapsulation percentage of each compound was calculated by evaluating the area of each peak present in LC-MS chromatographs. Table 3 and Table 4 summarize the characteristic compounds which are derived through LC/MS [ESI(+)] and LC/MS [ESI(-)] determination, respectively, as well as the percentage of their encapsulation. It is evident that by applying different ionization conditions, different compounds are detected. Results revealed a dependence between the polymeric percentage, which was utilized during the nanoparticles preparation, and the encapsulation of the compounds which are present in the PJ. More specifically, the encapsulation is slightly elevated when 5.0 wt% of the polymeric material was utilized contrary to the 2.5 wt% samples in both derivatives. This was expected since by increasing the solution concentration of CS or CS-SDAEM, more mass and reactive groups are available to interact with PJ compounds and to encapsulate them by absorption. However, in most of the cases the differences are very small in order to extract any clear conclusion. The same also appears when comparing the effect of the used polysaccharide type. In all nanoparticles formed by CS or CS-SDAEM, the entrapment percentage reveals no significant differences. Interesting is the fact that hydrophilic compounds are encapsulated at higher percentage, compared to the hydrophobic ones. From these data it is apparent that several acids and lots of the phenolic compounds existed in PJ, have been encapsulated in high percentages to the prepared nanoparticles [71]. These compounds are well known to have high antioxidant properties and PJ is a natural source containing them in a high rate [72,73].

### 3.3. Characterization of the Emulsions

According to the above studies, it is clearly demonstrated that the PJ-encapsulated nanoparticles prepared with polymer solution concentrations 2.5 wt%, have the lowest sizes and also similar encapsulation percentage of PJ bioactive compounds with the nanoparticles prepared using 5 wt% polymer concentrations. For these reasons in a further step, the prepared CS-TPP-PJ_2.5 wt% and CS-SDAEM-TPP-PJ_2.5 wt% nanoparticles have been chosen to be added to O/W emulsions. The emulsions were consisted of 75% water phase and 25% oil phase and in order to evaluate the effect of nanoparticles on emulsion properties and their stability during storage, different concentrations of PJ encapsulated nanoparticles like 0.7, 1.4, 2.1 and 2.9% *w*/*v* were added. For comparison purposes, a blank sample without the addition of encapsulated PJ nanoparticles was prepared. 

**Figure 10:**
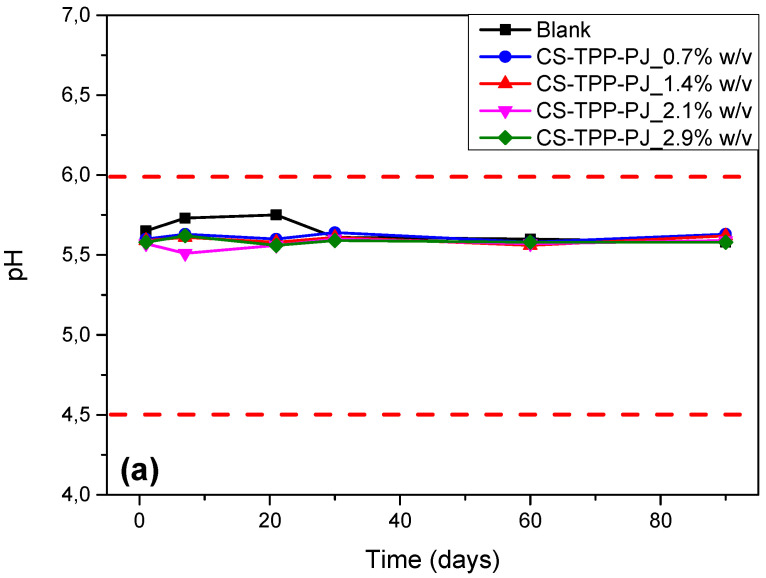

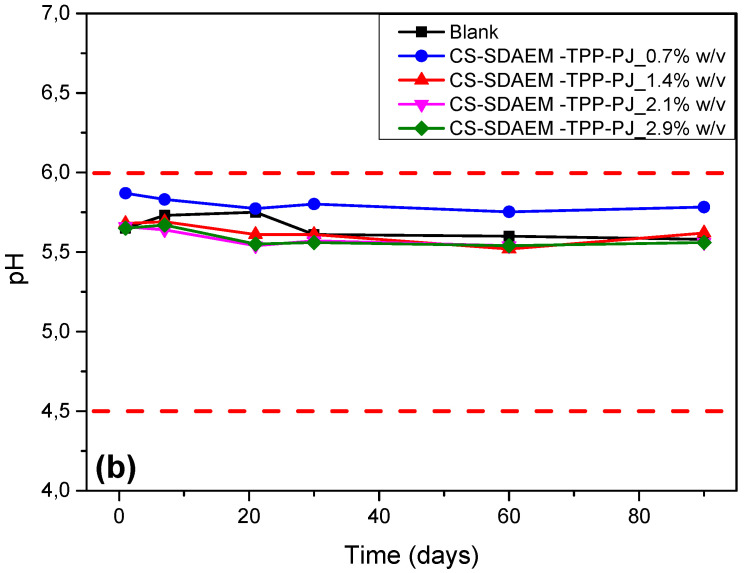
pH values of the prepared emulsions during storage stability studies of the samples (**a**) CS-TPP-PJ_0.7, 1.4, 2.1 and 2.9% *w*/*v* and (**b**) CS-SDAEM-TPP-PJ_0.7, 1.4, 2.1 and 2.9% *w*/*v*. The red dotted lines depict the pH of the healthy human skin.

The examination of the pH values of the prepared samples is of crucial importance since extreme values might possibly occur various skin reactions. The pH of the healthy human skin varies between 4.5–6. Consequently, the pH of the prepared O/W emulsions should be in between this region [74]. Figure 10a,b depict the pH values of the prepared emulsions, which varies from 5.5 to 6.1. From these figures it is clear that all emulsions have pH values similar to the blank sample and at the same time good pH stability for up to 90 days. In almost all cases the addition of CS-TPP-PJ_2.5 wt% and CS-SDAEM-TPP-PJ_2.5 wt% nanoparticles at different amounts (0.7 up to 2.9 *w*/*v*), does not seem to affect the pH values. This was expected since the added amounts were too small. The constant pH values observed over a period of time is a factor contributing to the stability of the emulsion. So, from these measurements it can be concluded that all emulsions are likely to have steady pH values during storage, with no significant variations, making them suitable for cosmetic dermal applications. 

The stability of the prepared O/W emulsions was confirmed via centrifugation test. Gravitational separation of emulsions is a phenomenon observed in various commercially available products. Table 5 confirms that no phase separation was observed at any sample during the first 7 days of study. According to Smaoui et al., this observation is attributed to proper homogenization speed of the emulsion during the experimental procedure [75]. 

**Figure 11:**
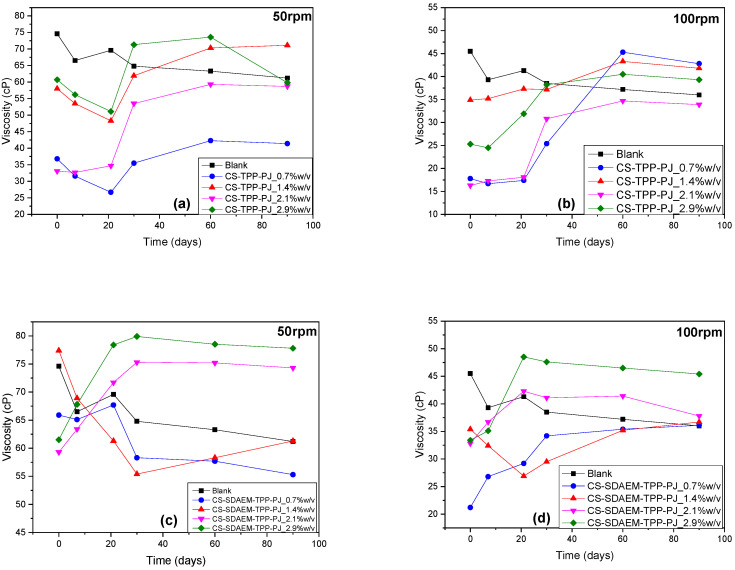
Viscosity values of the prepared emulsions during 90 days (**a**) CS-TPP-PJ_0.7, 1.4, 2.1 and 2.9% *w*/*v* 50 rpm, (**b**) CS-TPP-PJ_0.7, 1.4, 2.1 and 2.9% *w*/*v* 100 rpm (**c**) CS-SDAEM-TPP-PJ_0.7, 1.4, 2.1 and 2.9% *w*/*v* 50 rpm and (**d**) CS-SDAEM-TPP-PJ_0.7, 1.4, 2.1 and 2.9% *w*/*v* 100 rpm.

Viscosity variation is an important factor concerning O/W emulsions as it is an indication of their inner stability and moreover, it affects the appealing of the final product to the consumers. Defects in the viscosity of the final formulations would potentially provoke phase separation and liquefaction. For the improvement of the long term stability of the prepared emulsions as well as the increase of their viscosity during storage, the addition of acrylic polymers in the aqueous phase of O/W emulsions has been proposed [76]. Furthermore, it was found that the addition of various nanoparticles during storage results in an augmentation of the emulsion’s viscosity. Pei et al. studied the influence of silica nanoparticles and the reported results confirmed that these nanoparticles contribute to an increased long term stability of studied emulsions [77]. Figure 11 a–d presents the viscosity values of the emulsions containing PJ nanoparticles for up to 90 days with 50 and 100 rpm measurements. Concerning the blank samples, there is a progressive yet very small reduction of viscosities during storage, which is attributed to the necessary time of the emulsions to coagulate. The addition of nanoparticles causes in all cases a reduction of the emulsion viscosities, as was expected, since these are solid particles. However, after 20–30 days the viscosities have been increased in all emulsions reaching values very close to these of the blank emulsion. After this period of time, the emulsions’ viscosities are stable and this is due to the hydration of nanoparticles, which results to the stability of the final emulsions. However, in the case of CS-TPP-PJ emulsions, at rotation speed 50 rpm, there is no clear effect of the added amounts of nanoparticles to the viscosity values. Values are completely different for emulsions prepared with CS or CS-SDAEM nanoparticles. In the case of CS-TPP-PJ emulsions, those with 1.4 and 2.9 *w*/*v* have the highest viscosities, while concerning CS-SDAEM-TPP-PJ formulation, the emulsions with 2.1 and 2.9 have the highest viscosities. This was expected due to the addition of solid nanoparticles to W/O emulsions. On the contrary, when the emulsions studied at rotation speed 100 rpm, there is a better correlation of measured viscosities with added amount of nanoparticles. Apparently, in both cases (CS-TPP-PJ or CS-SDAEM-TPP-PJ) the emulsions with the highest amount of nanoparticles (2.9 *w*/*v*) have the highest viscosities, followed by these with 2.1 or 1.4 *w*/*v*. Furthermore, it can be seen that emulsions with 1.4 and 2.1 *w*/*v* nanoparticles, have viscosities very close to that of blank emulsion with small differences and maybe they are the best for the preparation of final emulsions. 

Pomegranate has been also studied concerning its sun-protective ability. Table 4 presents the SPF values of the prepared emulsions along with the blank one. The values are calculated according to the Equation (2)**,** and the absorption values were obtained in the range of 290–320 nm. All samples had SPF factor values varying from 1.86–2.9. The differences between CS-TPP-PJ and CS-SDAEM-TPP-PJ nanoparticles are too small, which is an indication that the polymer matrix has negligible effect on SPF index. However, it seems that the added amount has some effect. The emulsions with 0.7% *w*/*v* nanoparticles had the smallest values, while above this amount there is a stabilization, even though emulsions with 2.9 *w*/*v* had slightly highest SPF values. As expected, due to the presence of the PJ loaded CS nanoparticles, all the emulsions appear to have improved SPF values compared to the blank sample. According to FDA (U.S. Food and Drug Administration), any sunscreen agent utilized in sunscreen emulsions should display a minimum of SPF 2, and it is clear that all studied emulsions with nanoparticles added in an amount larger than 1.4%*w*/*v*, meet this requirement. The addition of encapsulated PJ nanoparticles certainly contributes to this high SPF values and similar findings have been reported in literature. Ranjithkumar et al., studied the effect of pomegranate seed oil on the protection of the skin from the UV exposure and results indicated sufficiently SPF ability of the pomegranate oil [78]. Furthermore, Afaq et al., confirmed the photochemopreventive effect of the pomegranate derived products [79] while in a recent study it was reported that pomegranate extract and pomegranate juice consumption resulted in an increase in skin protection to UVB exposure [80]. In both cases by increasing the minimal erythema dose a decrease to melanin formation was fond, indicating an enhancement of UVB protection. In previous in vitro and animal studies the topical application of pomegranate and EA improved the resistance of skin to UVB exposure [81], as well as after in vitro studies in human skin fibroblasts, keratinocytes or reconstituted skin [82,83]. The UVB photoprotection of PJ on the skin include oxidation, inflammation, melanin formation, apoptosis of keratinocytes, activity of matrix metalloproteinases, collagen and elastin formation and this is due to the anthocyanin compounds that PJ contains [84]. Consequently, the increase of the SPF emulsions’ values in our study it is attributed to the presence of the encapsulated PJ. Such a UV protection increase was also found in similar cosmetic emulsions prepared from encapsulated annatto and saffron in CS nanoparticles [34]. 

CS has been chosen for nanoencapsulation of PJ since it has some antimicrobial activity [45,46]. From many studies it was found that among our advantages, pomegranate juice also has potent antibacterial and antifungal properties due to polyphenols as well as due to the ellagic and gallagic acid, punicallins and punicalagins [85,86]. These phenolic compounds exhibit synergistic effects towards different microorganisms [87]. Thus, by adding PJ to emulsions, it is expected to enhance their antibacterial properties and effectively replace synthetic and chemical antibacterial additives that occur naturally. The antibacterial properties of the prepared emulsions against *E. coli* and *S. aureus* have been studied in comparison with neomycin, which is a compound extensively used in cosmetic emulsions. As can be seen from Table 6, neat CS has some antibacterial properties against both bacteria that were slightly higher in the CS-SDAEM derivative due to its quaternary ammonium groups.

The addition of CS-TPP-PJ nanoparticles produces emulsions with higher inhibition zones than neat CS in all concentrations close to that of neomycin. However, the results for the emulsions containing CS-SDAEM-TPP-PJ, showed inhibition zones even higher than that of neomycin when PJ loaded nanoparticle concentrations of 2.1% and 2.9% were used. This is due to the synergistic effect of the polymer matrix and the antibacterial activities of the PJ compounds. Furthermore, from Table 6 it is clear that the recorded inhibition zones in all emulsions (9–14 mm) are slightly broadened by increasing the added amount of nanoparticles in emulsions and are very close to that of neat PJ reported in literature. Shivsharan et al., reported that pomegranate juice has high antimicrobial activities and a maximum inhibition zone of 12 mm against *E.coli* [88]. Similar inhibition zones were also reported in another study, which directly depended on the used concentration of aqueous extract of pomegranate juice [89]. In both *Staphylococcus aureus* and *Escherichia coli* the inhibition zones increased from about 8.5 up to 14 mm, by increasing the aqueous extract concentration from 10 mg/dL up to 30 mg/dL, respectively. These are in well agreement with previous reported studies [90]. In addition, minimal inhibitory concentration (MIC) ranging from 0.05 to 0.20 mg/mL has also been reported by Lantzouraki et al. [91]. From to these studies it is clear that the nanoencapsulation of PJ seems to be an effective method to encapsulate the active compounds of PJ, which are responsible for its antibacterial properties. In this study, this can be seen in Table 6 but also a complete characterization of these properties has been reported by Lantzouraki et al. [91]. Due to their high antibacterial activity, pomegranate pericarp extracts have been reported to enhance the antibacterial activity of ciprofloxacin, which is a well-known and extensively used antibiotic drug [92].

**Figure 12:**
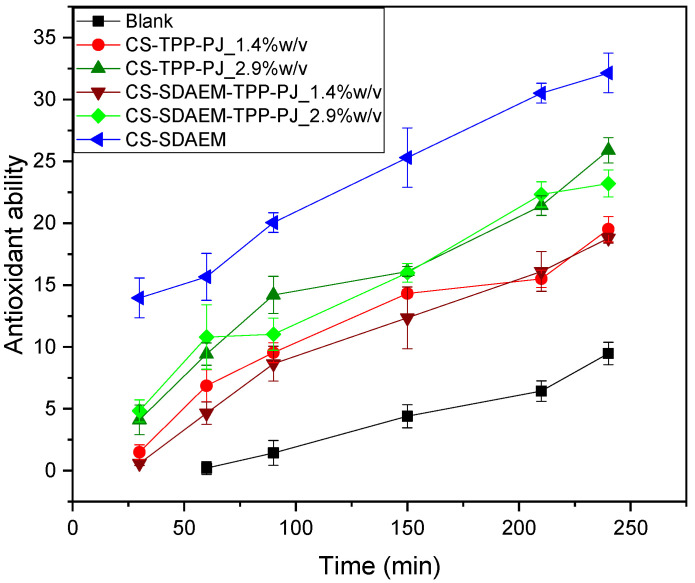
Study of the kinetic of the antioxidant ability of emulsions during the first 4 h.

From the results mentioned above, it is clear that all samples exhibit enhanced SPF and antibacterial activities and were further evaluated for their antioxidant ability. Lipid oxidation is the main cause that engenders demotion of O/W emulsions’ quality, leading to its demulsification and quality deterioration [93]. Therefore, it is mandatory to assess the antioxidant activity of prepared emulsions and for this reason several antioxidants and protectants are used. There are many natural sources of antioxidants and among these, pomegranate fruit was found to be effective in retarding the process of lipid oxidation [94,95]. This was also tested in the present work. Figure 12 demonstrates the kinetic behavior of the antioxidant ability of the emulsions during the first 240 min. It depicts the gradual augmentation of the antioxidant activity of the emulsions containing both CS and CS-SDAEM PJ loaded nanoparticles. Notable is also the fact that all samples reveal enhanced antioxidant ability compared to the blank sample, while the increase of the added percentage of CS or CS-SDAEM nanoparticles containing PJ, results in a slightly augmented improvement of the emulsions’ antioxidant activity. This trend predominantly is attributed to the presence of the phenolic compounds of the PJ, clearly indicating a correlation between the added amount of the PJ nanoparticles and the antioxidant activity of the emulsions. Similar antioxidant effect of PJ were also recently reported by Les et al. [96] which has been attributed to antioxidant polyphenols, including ellagitannins (hydrolysable tannins) and anthocyanins (condensed tannins) [23,85]. Furthermore, it was found that some of these compounds have higher antioxidant activity than others following the order: “Porphiroyeneti” > “Wonderful” > “Persephone” [91]. Moreover, since the sample CS-SDAEM-TPP-PJ reveals supreme antioxidant activity percentage compared to CS-TPP-PJ, the boost on the antioxidant activity of the nanoparticles in the final O/W emulsion is due to the use of CS-SDAEM derivative.

## 4. Conclusions

In the present study, CS was successfully modified with the zwitterionic monomer SDAEM, and PJ was encapsulated into CS and CS-SDAEM nanoparticles via ionic gelation technique. The successful modification of CS structure was confirmed by FTIR and ^1^H-NMR spectra through the new formed bond between the amino group of CS and the methylene group of the monomer. XRD pattern revealed the reduced crystallinity of the new CS-derivative compared to neat CS. PJ loaded nanoparticles of neat CS as well as of CS-SDAEM were successfully prepared. DLS measurements revealed their actual size which varied between 426 nm and 4.5 μm depending on the experimental conditions and mainly the used polymer solution concentration. In addition, CS-SDAEM formed nanoparticles with lower sizes in all studied concentrations. FTIR revealed the formation of hydrogen bonds between the PJ and the polymeric matrixes and XRD patterns showed reduced crystallinity of PJ encapsulated nanoparticles, compared to the initial materials. Furthermore, the loaded ingredients of the PJ were detected and quantified via LC-MS with positive and negative ionization conditions. Finally, the prepared PJ-loaded nanoparticles with 2.5 wt% polymer solutions were chosen for the preparation of O/W cosmetic emulsions, since it was the optimum concentration that provided the lower particle sizes. Viscosity and pH measurements demonstrated good storage stability for up to 90 days at room temperature for all samples. The sun-protective ability of the emulsions was estimated by their SPF values which varied between 0.4 and 2.86 and is attributed to the anthocyanin compounds that PJ contains. Finally, the antibacterial activity of all prepared emulsions was enhanced, and this was due to the existence of PJ bioactive phenolic and other acid compounds, which are also responsible for the improved antioxidant properties of the formulations. All the above results show that PJ encapsulation in CS and CS-SDAEM derivative is a proper technique to enclose the bioactive compounds of PJ into the matrix and can be used for the preparation of cosmetic emulsions to enhance their properties. The addition of CS-SDAEM nanoparticles produces emulsions with valuable antibacterial and antioxidant properties, which is an additional advantage of this derivative over neat CS. 

## Figures and Tables

**Table 1:** List of ingredients used in the preparation of the emulsions (Water and Oil phase).

Ingredient		Samples			
	Blank	CS or CS-SDAEM-TPP-PJ_0.7	CS or CS-SDAEM-TPP-PJ_1.4	CS or CS-SDAEM-TPP-PJ_2.1	CS or CS-SDAEM-TPP-PJ_2.9
	Amount in Water Phase				
Water	140 g	139 g	138 g	137 g	136 g
Glycerin	7 g	7 g	7 g	7 g	7 g
Citric acid	1 g	1 g	1 g	1 g	1 g
Xanthan gum	2 g	2 g	2 g	2 g	2 g
Nanoparticles	-	1 mL	2 mL	3 mL	4 mL
	Amount in Oil Phase				
Olive Oil	26 g	26 g	26 g	26 g	26 g
Cetyl alcohol	4 g	4 g	4 g	4 g	4 g
Cetostearyl alcohol	4 g	4 g	4 g	4 g	4 g
Polysorbate 60	4 g	4 g	4 g	4 g	4 g
Shea butter	4 g	4 g	4 g	4 g	4 g
Steatic acid	4 g	4 g	4 g	4 g	4 g
Beeswax	4 g	4 g	4 g	4 g	4 g

**Table 2:** Nanoparticles size, polydispersity index (PDI) and zeta-potential results.

Sample	Z-Average (d.nm)	PDI	Zeta Potential (mV)
CS-TPP-PJ 2.5 wt%	647 ± 37	0.4	+23.4
CS-TPP-PJ 5.0 wt%	2403 ± 230	0.7	+29.1
CS-TPP-PJ 7.5 wt%	3530 ± 340	0.7	+35.2
CS-TPP-PJ 10.0 wt%	4556 ± 410	0.9	+35.4
CS-SDAEM-TPP-PJ 2.5 wt%	339 ± 21	0.3	+24.7
CS-SDAEM-TPP-PJ 5.0 wt%	426 ± 35	0.7	+26.6
CS-SDAEM-TPP-PJ 7.5 wt%	528 ± 42	0.6	+36.0
CS-SDAEM-TPP-PJ 10.0 wt%	745 ± 66	0.4	+33.5

**Table 3:** Compounds of PJ and their encapsulation efficiency in CS and CS-SDAEM nanoparticles via positive ionization LC/MS [ESI (+)].

Compounds	[M + H]^+^ *m*/*z*	% of Encapsulation of Compound			
		CS-TPP-PJ 2.5 wt%	CS-TPP-PJ 5.0 wt%	CS-SDAEM-TPP-PJ 2.5 wt%	CS-SDAEM-TPP-PJ 5.0 wt%
Pelargonidin-3-glucoside	433	90	91	81	92
Cyanidin-3-glucoside	449	89	96	78	88
Pelargonidin-3,5-diglucoside	595	70	79	64	74
Cyanidin-3,5-diglucoside	611	53	56	57	63
Delphinidin-3,5-diglucoside	627	98	96	80	85
(epi)gcat-cyd-3,5-dihexoside	815	64	70	63	74

**Table 4:** Compounds of PJ and their encapsulation efficiency in CS and CS-SDAEM nanoparticles via negative ionization LC/MS [ESI (-)].

Compounds	[M + H]^+^ *m*/*z*	% of Encapsulation of Compound			
		CS-TPP-PJ 2.5 wt%	CS-TPP-PJ 5.0 wt%	CS-SDAEM-TPP-PJ 2.5 wt%	CS-SDAEM-TPP-PJ 5.0 wt%
Ascorbic acid	175	61	82	89	90
Citric acid	191	99	98	61	71
L-malic acid	133	89	85	52	60
Chlorogenic acid	353	74	77	75	84
Ellagic acid	302	40	47	57	61
Coumaric acid-hexoside	325	76	83	80	82

**Table 5:** Physical characterization of the emulsions after centrifugation.

Sample	0 h	1 day	3 days	7 days
CS-TPP-PJ_0.7% *w*/*v*	-	-	-	-
CS-TPP-PJ_1.4% *w*/*v*	-	-	-	-
CS-TPP-PJ_2.1% *w*/*v*	-	-	-	-
CS-TPP-PJ_2.9% *w*/*v*	-	-	-	-
CS-SDAEM-TPP-PJ_0.7% *w*/*v*	-	-	-	-
CS-SDAEM-TPP-PJ_1.4% *w*/*v*	-	-	-	-
CS-SDAEM-TPP-PJ_2.1% *w*/*v*	-	-	-	-
CS-SDAEM-TPP-PJ_2.9% *w*/*v*	-	-	-	-

- = no phase separation, + = phase separation.

**Table 6:** Sun protection factor (SPF) values, standard deviation (STD) and Antibacterial activity of the prepared emulsions and inhibition growth zones against *E.coli* and *S.aureus*.

Sample	SPF Value	Area of Inhibition (diameter in mm)	
		*E. coli*	*S. aureus*
Neomycin	Not tested	12	10
CS	Not tested	6	7
CS-SDAEM	Not tested	8	11
Blank	1.68 ± 0.04	-	-
CS-TPP-PJ_0.7% *w*/*v*	1.89 ± 0.07	10	9
CS-TPP-PJ_1.4% *w*/*v*	2.49 ± 0.06	11	10
CS-TPP-PJ_2.1% *w*/*v*	2.40 ± 0.09	11	10
CS-TPP-PJ_2.9% *w*/*v*	2.90 ± 0.08	10	11
CS-SDAEM-TPP-PJ_0.7% *w*/*v*	1.86 ± 0.05	10	11
CS-SDAEM-TPP-PJ_1.4% *w*/*v*	2.67 ± 0.07	12	13
CS-SDAEM-TPP-PJ_2.1% *w*/*v*	2.54 ± 0.06	11	14
CS-SDAEM-TPP-PJ_2.9% *w*/*v*	2.82 ± 0.08	12	14
